# Clinicopathological Characteristics and Molecular Phenotypes of Primary Hepatic Lymphoma

**DOI:** 10.3389/fonc.2022.906245

**Published:** 2022-06-27

**Authors:** Ai-Yan Xing, Xin-Zhe Dong, Liu-Qing Zhu, Long Liu, Dong Sun, Sen Guo

**Affiliations:** ^1^ Department of Pathology, Shandong University Qilu Hospital, Jinan, China; ^2^ Department of Radiation Oncology, Shandong University Qilu Hospital, Jinan, China; ^3^ Nanjing Geneseeq Technology Inc., Nanjing, China; ^4^ Department of General Surgery, Shandong University Qilu Hospital, Jinan, China

**Keywords:** primary hepatic lymphoma, diffuse large B-cell lymphoma, gene features, next-generation sequencing, liver

## Abstract

Primary hepatic lymphoma (PHL) is a rare malignant tumor, occurring in 0.016% of non-Hodgkin’s lymphoma (NHL). The common histological subtype is diffuse large B-cell lymphoma (DLBCL). Due to the rarity of tumor, clinicopathological characteristics and molecular phenotypes of PHL are limited. Seven patients with PHL (primary liver DLBCL) and 13 cases of liver involvement by DLBCL diagnosed between 2014 and 2021 in our hospital were included. The genetic features were also compared between the two groups by next-generation sequencing (NGS). Differential gene expression and pathway enrichment analysis were also performed. There were some discrepancies on presenting symptoms, pathological characteristics, laboratory data, and prognosis between PHL and DLBCL-liver groups. No same mutation was found between PHL and DLBCL-liver groups by NGS. Differential gene expression analysis discovered some up- and downregulated genes in PHL compared with the DLBCL-liver group. Upregulated genes were enriched in metabolic pathways, and downregulated genes were enriched in the HTLV-1 infection pathway. PHL is a distinct entity, with unique molecular features compared to liver involvement of systemic lymphoma. Kaplan–Meier analysis showed that the prognosis of the PHL group was better than that of the DLBCL-liver group. Understanding the clinicopathological and molecular features of PHL would help to direct clinical treatment.

## Introduction

Primary hepatic lymphoma (PHL) is a very rare tumor accounting for 0.1% of all liver malignant tumors (1). For non-Hodgkin’s lymphoma (NHL), it constitutes about 0.016% of all NHLs and 0.4% of all primary extranodal NHLs (2). PHL is generally considered as a proliferative disease of lymphoid tissue that originates from intrahepatic lymphatic and residual hematopoietic tissue, and is characterized by no extrahepatic infiltration (3). Liver involvement is also common and present in more than 50% of cases of lymphoma (4). However, the prognosis of PHL patients is better than that of systemic lymphoma with liver involvement (5). We speculated that the clinical features and molecular phenotypes of PHL might be different, compared with liver involvement by lymphoma. Because of the rarity, there is a challenge on the diagnosis and therapy of PHL. The investigation of clinicopathological features and molecular phenotypes of PHL might have an important value on clinical management.

Diffuse large B-cell lymphoma (DLBCL) has been identified as the main type of PHL (6). Most reports of PHL published are mainly case reports, and there are few reports on the molecular characteristics of PHL at present. In this study, 7 cases of PHL (all were primary liver DLBCL) and 13 cases of liver involvement by DLBCL (DLBCL-liver) were analyzed retrospectively between 2014 and 2021 in our hospital. The pathological characteristics, etiologic factors, treatment, and follow-up were collected and compared between PHL and DLBCL-liver groups. We also attempted to reveal the discrepancy of molecular phenotypes between the two groups by NGS, which may help to facilitate an accurate diagnosis and to identify tumor biomarkers for contemporary systemic therapies of PHL.

## Materials and Methods

### Patients and Tissue Specimens

DLBCL was diagnosed according to the morphology and immunohistochemistry criteria by two pathologists in Shandong University Qilu Hospital between 2014 and 2021. Among them, 7 patients were described as having liver primary lymphoma at presentation with no involvement of spleen, lymph node, bone marrow, or other lymphoid tissues. The remaining 13 cases had DLBCL involvement of liver and extrahepatic tissue, such as spleen or lymph nodes. Clinicopathological information of the 20 patients was available from pathological records and patient files, including age, sex, presenting symptoms, laboratory examination, imaging, therapeutic regimens, and follow-up durations. Informed consents of all patients were obtained. The Ethics Committee of Shandong University approved this study, and the procedures involving human subjects were in accordance with the Declaration of Helsinki.

### Nucleic Acid Extraction and Library Construction

Genomic DNA and RNA were extracted from tumor FFPE samples using the QIAamp DNA FFPE Tissue Kit and the miRNeasy FFPE Kit, respectively. The fragment DNA was generated with Bioruptor (Diagenode, Bioruptor UCD-200). Ribosomal RNA was removed using RNase H followed by library preparation using the KAPA Stranded RNA-seq Kit with RiboErase (HMR) (KAPA Biosystems). Libraries were constructed using the KAPA Hyper DNA Library Prep Kit (KAPA Biosystem, KK8504). The dual-indexed sequencing libraries were PCR amplified with KAPA HiFi Hot start-ready Mix (KAPA, KK2602) for 4–6 cycles, then cleaned up by purification beads (Corning, AxyPrep Fragment Select-I kit, 14223162). Library concentration and quality were determined by the Qubit 3.0 system (Invitrogen) and Bioanalyzer 2100 (Agilent, Agilent HS DNA Reagent, 5067-4627), respectively.

### Hybrid Selection and Next-Generation Sequencing

The probes for targeted sequencing covered exons and selected introns of 475 leukemia- and lymphoma-related genes and were produced by Nanjing Geneseeq Biotechnology (Nanjing, China). RNA sequencing was performed by whole transcriptome RNASEQ detection (30M). The capture probes were added to the pre-library mixture in 5 min, and the solution hybridization was performed for 16–18 h at 65°C. After hybridization was completed, the captured targets were selected by pulling down the biotinylated probe/target hybrids using streptavidin-coated magnetic beads, and off-target library was removed using wash buffer. Then, the samples were purified by AMPure XP beads, quantified by qPCR (Kapa), and sized on Bioanalyzer 2100 (Agilent, Agilent HS DNA Reagent, 5067-4627). Libraries were normalized to 2.5 nM and were sequenced as paired 150-bp reads on Illumina HiSeq 4000 according to the manufacturer’s instrument.

### Sequencing Data Processing

Base calling was performed on bcl2fastq v2.16.0.10 (Illumina, Inc.) to generate sequence reads in FASTQ format (Illumina 1.8+ encoding). High-quality reads were mapped to the human genome (hg19, GRCh37 Genome Reference Consortium Human Reference 37) using the BWA aligner 0.7.12 with the BWA-MEM algorithm with default parameters to create SAM files. Picard 1.119 was used to mark duplicate reads by generating BAM files that were then sorted according to chromosome coordinates. The Genome Analysis Toolkit (GATK, version 3.4-0) was used to locally realign the BAM files at intervals with indel mismatches and recalibrate base quality scores of reads in BAM files.

### Differential Gene Expression Analysis

Quality control was performed with Trimmomatic (version 0.33 (7) STAR (version 2.5.3a) (8) was used for transcriptome mapping followed by isoform- and gene-level quantification, which was performed by RSEM (version 1.3.0 (9). Differential expression analysis was conducted by R packages DESeq2 (version 1.16.1 (10) and edgeR (version 3.18.1 (11) Differentially expressed genes were selected by fold change > 2 and *p*-value < 0.05. Corresponding volcano plots and heatmaps were generated by in-house R scripts. Gene Ontology (GO) and Kyoto Encyclopedia of Genes and Genomes (KEGG) enrichment analysis were performed by ClusterProfiler (version 3.4.4 (12) Unpaired, two-tailed Student’s *t*-test was used to determine differences between two groups. *p* < 0.05 was considered statistically significant.

### Statistical Analysis

Fisher’s exact probability tests were used for between-group comparisons. Overall survival (OS) analyses were performed using Kaplan–Meier plots and log-rank tests. Cox proportional hazards regression analysis was used for the evaluation of prognostic factors and calculation of hazard ratios (HRs) along with their 95% confidence intervals (CIs). OS was defined from the date of being diagnosed to the date of death. *p* < 0.05 was considered statistically significant. We used R (version 3.6.1) and R Bioconductor packages for all the above-mentioned analyses.

## Results

### Clinicopathological Characteristics

**Figure 1:**
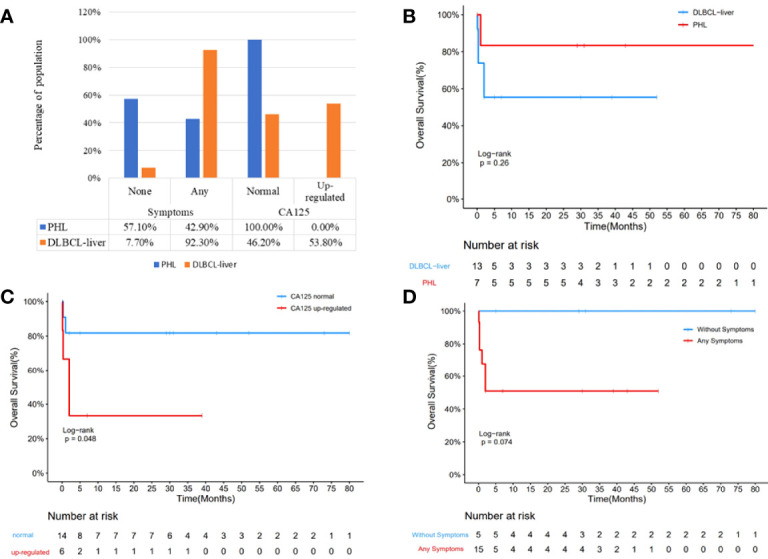
Comparison of clinical parameters of the PHL and the DBCLC-liver group. Clinical symptom was significantly different between the PHL and the DLBCL-liver group (**A**; *p* = 0.031). More upregulated CA125 was found in the DLBCL-liver group, although there is no significant difference (*p* = 0.051). Kaplan–Meier analysis showed that the OS of PHL was better than that of the DBCLC-liver group **(B)**. Compared with the CA125 upregulation group, the CA125 normal group showed better prognosis **(C)**. The longer survival of the without symptom group was observed compared with the symptom group **(D)**.

The clinicopathological characteristics of 7 patients with PHL and 13 patients with DLBCL-liver are summarized in **Table 1**. The median age of the patients in the two groups was 64 and 57 years, respectively (range, 31–78 and 29–71 years). Of the 7 patients with PHL, there were 4 male patients and 3 female patients. In the DLBCL-liver group, there were 10 male patients and 3 female patients. There were 4 patients (57.1%) with liver mass by health examination without any symptoms, and 3 patients (42.9%) with abdominal pain or distention in the PHL group. For the DLBCL-liver group, the common presenting symptoms were abdominal pain (8 cases; 61.5%), fever, and fatigue (4 cases; 30.8%). The remaining case showed no symptoms. The symptoms between the two groups showed statistical difference (**Figure 1A**; *p* = 0.031). Liver solitary or multiple masses were the main clinical image features of PHL, while liver and spleen (solitary or multiple) masses, hepatosplenomegaly, or lymphadenectasis were found in the DLBCL-liver group. As subtypes of DLBCL, the GCB (germinal center B cell) ratio and the non-GCB ratio were 4:3 and 11:2 in PHL and DLBCL-liver groups, respectively. A better prognosis of non-GCB was observed compared with the GCB subtype (**Supplementary Figure 1A**), which might be due to the fact that most non-GCBs were from PHL. The chemotherapy of the two groups was mainly the rituximab, cyclophosphamide, doxorubicin, vincristine, and prednisone (R-CHOP) regimen.

**Table 1:** The main clinical characteristics of 7 patients (Case nos. 1–7) with PHL and 13 patients with liver involvement of lymphoma (Case nos. 8–20).

Case no.	Age/sex	Symptoms	Clinical features	Pathology (DLBCL)	Treatment	HBV	AFP	CEA	ALT (U/L)	AST (U/L)	LDH (U/L)	CA125	Follow-up duration
1	39 F	Abdominal pain	Solitary mass	Non-GCB	Unknown	+	–	–	23	31	Unknown	–	Unknown
2	57 M	None	Solitary mass	GCB	R-CHOP×1 cycle	+	–	–	39	34	178	–	Alive, 80 months after diagnosis
3	68 M	None	Solitary mass	Non-GCB	Unknown	+	–	–	228	232	Unknown	–	Alive, 73 months after diagnosis
4	65 M	Abdominal distention	Solitary mass	Non-GCB	R-CHOP×2 cycles	+	–	–	35	30	184	–	Alive, 43 months after diagnosis
5	64 M	Abdominal distention	Multiple masses	GCB	R-COP×1 cycle	–	–	–	76	171	1,159	–	Die at 1 month after diagnosis
6	56 F	None	Solitary mass	Non-GCB	Unknown	–	–	–	459	650	673	–	Alive, 29 months after diagnosis
7	77 F	None	Solitary mass	GCB	R-CHOP×1 cycle	–	–	–	14	22	257	–	Alive, 31 months after diagnosis
8	48 M	Abdominal pain	Multiple masses	GCB	R-CHOP×6 cycles	–	–	–	13	45	460	446	Alive, 39 months after diagnosis
9	37 M	Abdominal pain	Solitary mass	GCB	R-CHOP×4 cycles	+	–	–	28	56	381	212	Die at 2 months after diagnosis
10	64 M	Fever, fatigue	Hepatosplenomegaly	GCB	Unknown	–	–	–	62	110	1,375	–	Die of 10 days after diagnosis
11	68 M	None	Solitary mass	non-GCB	R-CDOP×2 cycles R-CHOP×4 cycles	–	–	–	14	19	248	–	Alive, 5 months after diagnosis
12	57 M	Abdominal pain	Gastrointestinal hemorrhage	GCB	Unknown	+	–	–	114	1,151	Unknown	–	Unknown
13	44 F	Fever	Solitary mass	GCB	Unknown	–	–	–	154	120	1,587	294.9	Die at 2 days after diagnosis
14	29 F	Abdominal pain	Solitary mass	GCB	R-CHOP×4 cycles CHOP-E×4 cycles	–	–	–	28	45	1,084	–	unknown
15	50 M	Fever, fatigue	Multiple masses	GCB	R-CHOP×1 cycle	–	–	–	15	17	234	–	Alive, 52 months after diagnosis
16	57 M	Fever, fatigue	Hepatosplenomegaly	GCB	R-CHOP×6 cycles	+	–	–	30	21	178	70.9	Die at 2 months after diagnosis
17	71 M	Abdominal pain	Spleen solitary mass	GCB	Unknown	+	–	–	22	70	1,190	275	Die at 10 days after diagnosis
18	64 M	Abdominal pain	Lymphadenectasis	GCB	R-CHOP×4 cycles	–	–	–	22	19	250	–	Alive, 30 months after diagnosis
19	69 M	Abdominal pain and distention	Multiple masses	GCB	R-CHOP×6 cycles	+	–	–	128	140	834	2,266	Alive, 7 months after diagnosis
20	66 F	Abdominal pain, fever	Multiple masses	Non-GCB	R-CHOP×1 cycle	–	–	–	235	259	889	–	Alive, 2 months after diagnosis

Laboratory data revealed moderate to severe elevation of aspartate aminotransferase (AST) and alanine aminotransferase (ALT) in 3 patients (42.9%) in the PHL group, and in 5 patients (38.5%) in the DLBCL-liver group. By comparative analysis, we found that both the AST/ALT ≤ 1.5 and AST/ALT ≤ 2.0 groups might appear to have a better prognosis than the AST/ALT>1.5 and AST/ALT>2.0 groups (**Supplementary Figures 1B, C**), partly suggesting the important role of AST and ALT in DLBCL. As an important prognostic pretreatment parameter (13), serum lactate dehydrogenase (LDH) level was at an average of 490 U/L in the PHL and 725 U/L in the DLBCL-liver group, but was not statistically different (*p* = 0.3712). However, the longer survival of the LDH ≤ 300 U/L group was observed compared with the LDH>300 U/L group (**Supplementary Figure 1D**). Hepatitis B viral (HBV) infections were found in both PHL (4 cases; 57.1%) and the systemic groups (5 cases; 38.5%). It seems that there is a higher HBV-positive rate in the PHL group, but Fisher’s exact test showed that there is no significant difference between the two groups (*p* = 0.332) due to the small sample size. The α-fetoprotein (AFP) and the carcinoembryonic antigen (CEA) levels were normal in all patients tested in both groups. Additionally, upregulated CA125 was found in 6 patients (46.2%) in the DLBCL-liver group, but none in the PHL group (**Figure 1A**; *p* = 0.051).

Study date ends on October 31, 2021; in the PHL group, 1 patient died of hepatic encephalopathy. Except for 1 patient lost to follow-up, the survival time ranged from 1 to 80 months. In the DLBCL-liver group, 5 patients died of lymphoma. Moreover, 2 patients were lost to follow-up, and the survival time was 19 months (range, 10 days to 52 months). Though median survival time has not been reached, of the total of 20 cases, Kaplan–Meier analysis showed a better prognosis of the PHL group than the DLBCL-liver group (**Figure 1B**). In addition, the normal CA125 group and the without symptom group showed a longer survival time (**Figures 1C, D**). The log-rank analysis was also performed. The results showed that CA125 was an independent prognostic factor (**Supplementary Table 1**; *p* = 0.0438). We also found that the upregulated level of AST/ALT, pathology subtypes, symptom, and LDH might be potential indicators of poor prognosis.

### Landscape of Genomic Alterations in PHL

**Figure 2:**
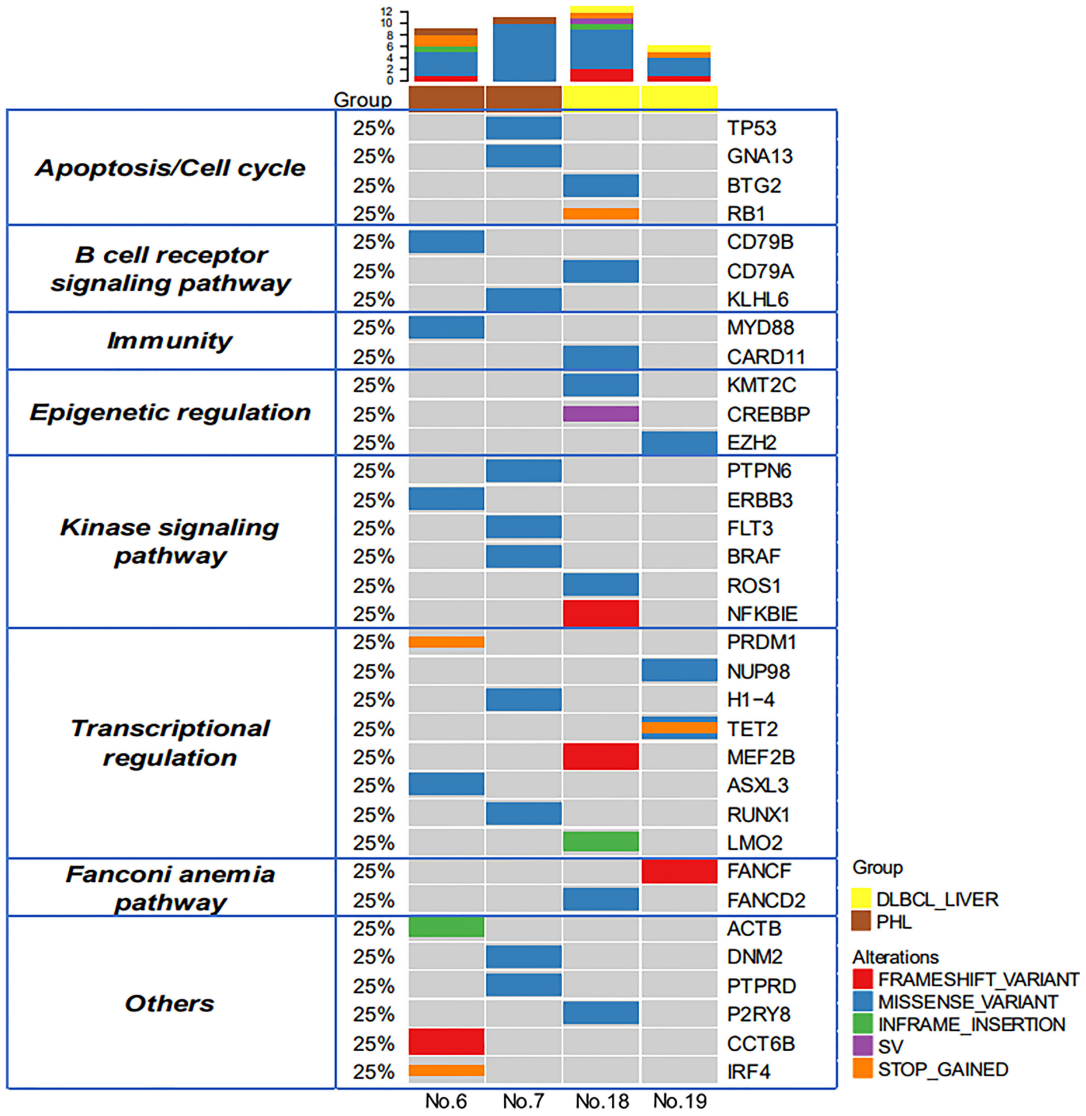
Genomic profiles of PHL and DCBCL-liver. Thirty-four putative driver genes and 35 alterations were identified between the PHL and the DCBCL-liver group. The upper bars represent the mutation number for each patient. The left table shows the apparent regulatory pathway to which the above genes belong.

Because of sample scarcity, 2 cases of PHL and 2 cases of DLBCL-liver were sequenced using DNA-based 475 genetic testing (1000X) and RNA-based whole transcriptome RNASEQ detection (30M). The distribution of somatic mutations of these 4 cases is shown in **Figure 2**. There were 34 putative driver genes and 35 alterations were identified. The mean number of mutations per case was 9.25. Signal pathway involvement by the mutations was mainly on apoptosis/cell cycle, the B-cell receptor signaling pathway, the kinase signaling pathway, and transcriptional regulation. However, the heterogeneity was obvious, without consistent mutations found between PHL and DLBCL-liver groups. It was worth noting that there were no mutations related to the apparent regulatory pathway and the Fanconi anemia pathway (DNA repair pathway) in PHL. This result partly suggested that the molecular profiles of PHL might be different from DLBCL-liver.

**Figure 3:**
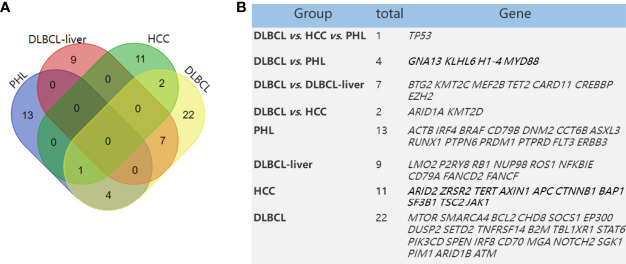
Mutated genes in PHL and DLBCL-liver compared with HCC and DLBCL in a previous study. Venn diagram demonstrates the number of altered genes shared in PHL, DLBCL-liver, HCC, and DLBCL **(A)**. The table shows corresponding numbers and gene names **(B)**.

Anupama et al. performed an integrative gene analysis of a cohort of 1,001 DLBCL patients by whole-exome and transcriptome sequencing (14). We also compared these high-frequency mutated genes of the above study with our panel and found that there were 36 mutated genes in our testing. Furthermore, we compared the differences in mutational genes among PHL, DLBCL-liver, and DLBCL. We found that the PHL and DLBCL groups shared 5 majority driver genes (**Figure 3A**), namely, TP53, GNA13, KLHL6, H1-4, and MYD88, and the DLBCL-liver and DLBCL groups shared 7 majority driver genes, namely, BTG2, KMT2C, MEF2B, TET2, CARD11, CREBBP, and EZH2; 27.8% (5/18) of the genes in PHL were mutated in DLBCL. This result demonstrated that PHL shared some molecular characteristics of DLBCL. Genetic sequencing might assist in the differential diagnosis of PHL.

### The Genetic Characteristics of PHL Are Obviously Different From Hepatocellular Carcinoma

The main presenting symptoms and clinical features of PHL are usually similar to HCC. There was a risk of misdiagnosing PHL as HCC. James et al. (15) have found some frequently mutated genes (mutation frequency >5%), such as TP53, ARID2, ZRSR2, TERT, AXIN1, APC, CTNNB1, BAP1, SF3B1, TSC2, and JAK1, in HCC compared with normal tissues by NGS. Of these 14 mutated genes in HCC, compared with mutant genes in PHL in this study (ACTB, IRF4, CD79B, DNM2, CCT6B, ASXL3, RUNX1, PTPN6, PRDM1, PTPRD, FLT3, ERBB3, and TP53), we found no other same mutant gene between the PHL and HCC groups except for the TP53 gene. TP53 is a tumor suppressor gene, whose mutation is closely related with the occurrence of many tumors. Thus, we concluded that the gene phenotypes of HCC and PHL are significantly different (**Figure 3B**).

### Specific Genes and Biological Pathways Were Enriched in PHL Compared to DLBCL-Liver

**Figure 4:**
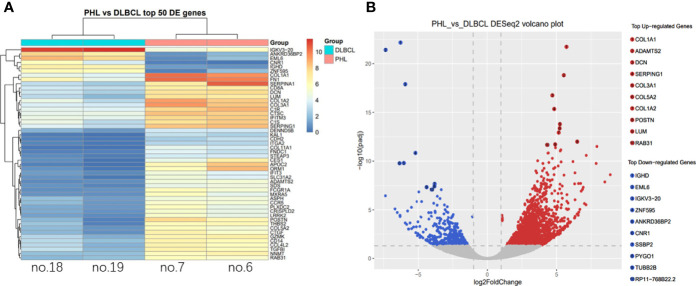
Differential expressed gene analysis. Differential expressed genes were shown between the PHL and the DCBCL-liver group by cluster analysis **(A)**. The column represents the sample; each row represents a gene; the right side is marked as the gene name; the color represents the expression, changing from red to blue as expression decreases. The top 10 up- and downregulated genes in PHL were showed compared with the DLBCL-liver group **(B)**. Each point in the volcano map represents a gene, and the red point is a significantly different gene that meets the thresholds of |log2FC| > 1 and *p*-value < 0.05. The blue dots are genes that only satisfy *p-*value < 0.05.

Differentially expressed genes were shown between the PHL and DCBCL-liver groups by cluster analysis (**Figure 4A**). Compared to the DLBCL-liver group, the top 10 upregulated genes in PHL were COL1A1, ADAMTS2, DCN, SERPING1, COL3A1, COL5A2, COL1A2, POSTN, LUM, and RAB31. The top 10 downregulated genes were IGHD, EML6, IGKV3-20, ZNF595, ANKRD36BP2, CNR1, SSBP2, PYGO1, TUBB2B, and RP11-768B22.2, both with identical statistical significance (*p* < 0.05, **Figure 4B**).

**Figure 5:**
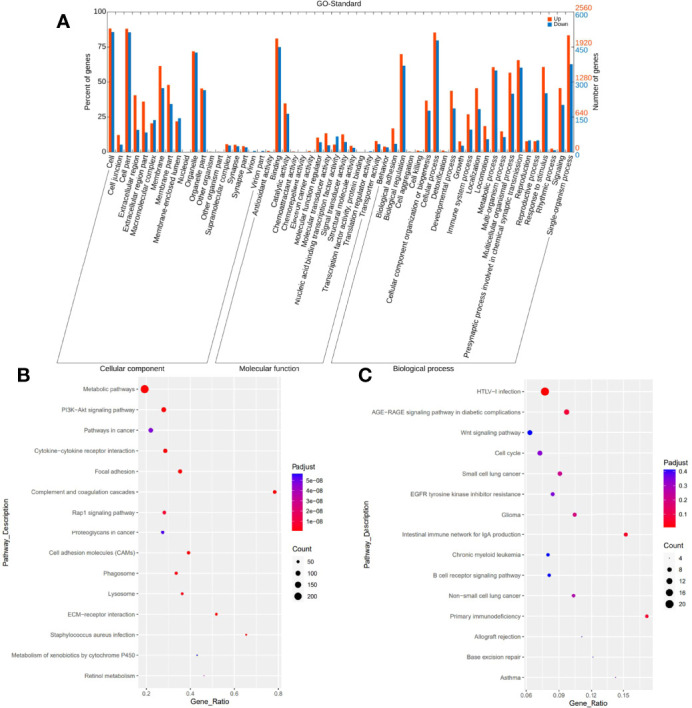
GO and KEGG pathway enrichment analysis. GO analysis shows upregulated and downregulated GO terms mainly involving cell, cell part, binding, cellular process, and signaling **(A)**. KEGG pathway enrichment analysis reveals upregulated genes mainly enriched in metabolic pathways, PI3K-AKT signaling pathway, pathways in cancer, cytokine–cytokine receptor interaction, and focal adhesion pathways **(B)**. Downregulated genes are more enriched in HTLV-I infection, AGE-RAGE signaling pathways in diabetic complications, Wnt signaling pathway, cell cycle, and small cell lung cancer pathways **(C)**.

Additionally, we conducted GO term, KEGG pathway enrichment, and differential gene expression analysis to see if specific genes are up- or downregulated and if those genes were enriched in specific GO terms and signaling pathways in PHL compared to the DLBCL-liver group. GO analysis showed the cellular component, molecular function, and biological process of these differentially expressed genes. The top 5 upregulated and downregulated GO terms involved cell, cell part, binding, cellular process, and signaling (**Figure 5A**). Pathway enrichment revealed that upregulated genes were more enriched in metabolic pathways, PI3K-AKT signaling pathway, pathways in cancer, cytokine–cytokine receptor interaction, and focal adhesion pathways (**Figure 5B**). Downregulated genes were more enriched in human T-cell lymphotropic virus type 1 (HTLV-1) infection, AGE-RAGE signaling pathways in diabetic complications, Wnt signaling pathway, cell cycle, and small cell lung cancer pathways (**Figure 5C**).

## Discussion

Because PHL is a rare tumor, little is known about its pathogenesis and molecular mechanism. In this study, we reviewed the clinicopathological characteristics and molecular phenotypes of 7 cases of PHL and 13 cases of DLBCL-liver. We aimed to find some discrepancy on pathological features and gene phenotype between these two diseases to guide clinical management.

PHL, as a proliferative disease of lymphoid tissue, is characterized by no extrahepatic infiltration. Currently, the diagnosis of PHL mainly depends on pathology and imaging. The early diagnosis of PHL is still difficult at present, especially distinguishing it from DLBCL-liver without biopsy. However, according to the results of our study, we need to consider the following possibilities for PHL in some conditions: no clinical symptoms but with liver masses, and no abnormal levels of serum AFP, CEA, and CA125, but slightly upregulated ALT/AST and LDH levels. As for DLBCL-liver, most patients have clinical symptoms, such as abdominal pain or distention, fever, and fatigue. Imaging reveals liver or spleen masses or multiple swollen lymph nodes. Levels of serum AFP and CEA are normal, but CA125, ALT/AST, and LDH levels could be obviously upregulated. The prognosis of patients with DLBCL-liver is worse than those with PHL. Our results suggest that a comprehensive test is necessary to diagnose patients with DLBCL-liver, if obvious symptoms and abnormal serum markers are observed. Circulating tumor DNA (ctDNA) detection is now used in a variety of tumors. It hopes that the role of ctDNA is defined on the auxiliary diagnosis of PHL and DLBCL-liver in the future.

At present, the overall prognosis of PHL is better, and there are few studies on the prognostic factors of PHL. Of the total of 20 cases, the log-rank analysis showed that CA125 was an independent prognostic factor. We also found that the upregulated levels of AST/ALT and LDH might be potential indicators of poor prognosis. These indicators are much higher in patient no. 5 of this study, the only patient who died in the PHL group. However, due to the small number of PHL samples, no detailed analysis was carried out. The clinician needs to pay more attention to these patients with higher levels of CA125, AST/ALT, and LDH.

DLBCL is a genetically heterogeneous malignant tumor, regardless of whether it has a liver origin or has a systemic involvement. Recent advancements in gene sequencing analysis have provided new opportunities for novel targeted therapy for patients with lymphoma (16). Thus, we further explored the molecular features of PHL and DLBCL-liver in order to reveal the discrepancy between the two groups at the genetic level. The DNA-based sequencing and RNA-based sequencing results showed that there were 18 mutations found in PHL and 17 mutations found in DLBCL-liver. No same mutation was observed between the two groups, perhaps due to the obvious tumor heterogeneity. However, compared with DLBCL-liver, no mutations related to the apparent regulatory pathway and the Fanconi anemia pathway (DNA repair pathway) were found in PHL, partly suggesting that the gene phenotype of PHL might be different from that of DLBCL-liver.

The mutated genes of PHL were also compared with the high-frequency mutated genes reported in HCC and DLBCL. Except for TP53, the mutated genes of PHL were significantly different from HCC, highlighting the possibility of differential diagnosis of PHL and HCC based on NGS. Five majority driver genes, namely, TP53, GNA13, KLHL6, H1-4, and MYD88, were shared in PHL and DLBCL groups, suggesting that PHL shared some molecular features with DLBCL. Thus, the similarities in therapeutic regimens of PHL and DLBCL were adopted by clinicians. More PHL cases need to be collected to further elaborate whether the mutation spectrum of PHL is different from DLBCL in the next study.

PHL showed distinct gene expression patterns compared to DCBCL-liver, although the pathology type is the same. Compared to DCBCL-liver, 10 upregulated genes (COL1A1, ADAMTS2, DCN, SERPING1, COL3A1, COL5A2, COL1A2, POSTN, LUM, and RAB31) and 10 downregulated genes (IGHD, EML6, IGKV3-20, ZNF595, ANKRD36BP2, CNR1, SSBP2, PYGO1, TUBB2B, and RP11-768B22.2) were detected in PHL. Many of these genes are also cancer-related. For example, COL1A1, reported as an oncogene, promotes metastasis in breast cancer (17) and colorectal cancer (18). The EML6-ALK fusion variant associates with crizotinib response in lung adenocarcinoma (19). Pathway enrichment revealed that upregulated genes are enriched in metabolic pathways, and downregulated genes are enriched in the HTLV-1 infection pathway. The genetic variations in metabolic pathways were reported to be predictable on prognosis and survival of NHL (20). HTLV-1 causes a lifelong infection and is associated with an aggressive form of leukemia (21). The distinct gene expression signature of PHL may not only aid differential diagnosis, but also provide insights into understanding the molecular pathways involved in PHL. However, there were some limitations in our research. First, due to the long history and scarcity of tissue samples, only 2 PHL and 2 DLBCL-liver samples were sequenced. We have not yet analyzed the correlation between the genetic changes and clinicopathological manifestations. Second, the pathogenic mechanisms of the above molecular phenotypes on PHL have not been further studied. More prospective studies are required to further verify the clinicopathological characteristics and molecular phenotypes of PHL.

In conclusion, we reported the clinicopathological characteristics of PHL in a Chinese population. PHL is a distinct entity, with unique molecular features compared to liver involvement of systemic lymphoma. The overall prognosis of PHL is relatively better after chemotherapy. Therefore, early accurate identification and individualized treatment are critical for clinical management.

## Data Availability Statement

The original contributions presented in the study are publicy available. This data can be found here: https://ngdc.cncb.ac.cn/gsa-human/, HRA002577. Further inquries can be directed to the corresponding author/s.

## Ethics Statement

The studies involving human participants were reviewed and approved by the Ethics Committee of Shandong University. The patients/participants provided their written informed consent to participate in this study.

## Author Contributions

A-YX and SG were the principal investigators and designed the research. A-YX, X-ZD, LL, L-QZ, and DS performed the experiments. A-YX, L-QZ, DS, and SG analyzed the results and wrote the manuscript. All authors contributed to the article and approved the submitted version.

## Funding

This work was supported by the Shandong Province Key R&D Program (No. 2021CXGC011105-7), the Beijing Medical Award Foundation (No. 6010121060), Chen Xiaoping Science and Technology Development Fundation (No. CXPJJH11800004-021) and the Natural Science Foundation of China (No. 81902884).

## Conflict of Interest

Author L-QZ is employed by Geneseeq Technology Inc.

The remaining authors declare that the research was conducted in the absence of any commercial or financial relationships that could be construed as a potential conflict of interest.

## Publisher’s Note

All claims expressed in this article are solely those of the authors and do not necessarily represent those of their affiliated organizations, or those of the publisher, the editors and the reviewers. Any product that may be evaluated in this article, or claim that may be made by its manufacturer, is not guaranteed or endorsed by the publisher.
