# Supplementary material for: Clinicopathological Characteristics and Molecular Phenotypes of Primary Hepatic Lymphoma
Source: Front Oncol. 2022 Jun 27;12:906245. doi: 10.3389/fonc.2022.906245 (PMC9272565; doi:10.3389/fonc.2022.906245)
Supplement: Supplementary file 1 [file DataSheet_1.docx]

Supplementary Figure 1


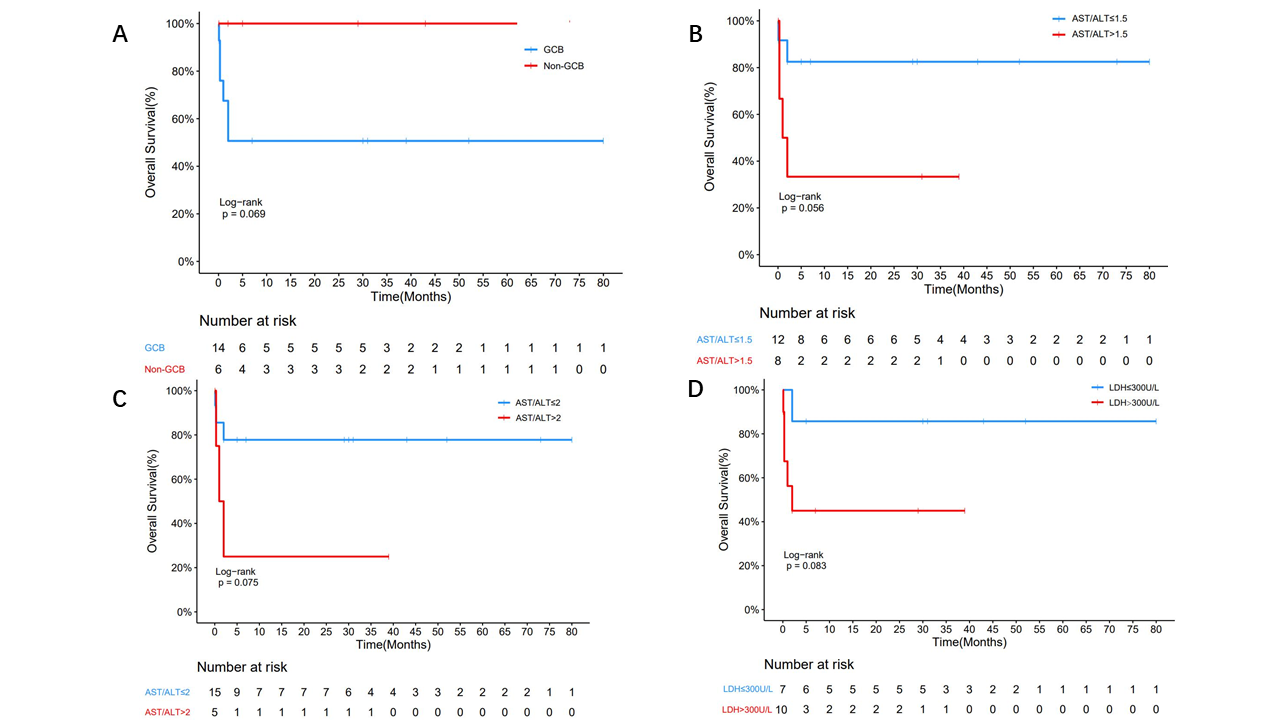


Kaplan–Meier analysis showed the overall survival of non-GCB group were better than GCB group (A). Compared with AST/ALT>1.5 and AST/ALT>2.0 group, AST/ALT≤1.5 and AST/ALT≤2.0 group showed better prognosis (B and C). And the longer survival of LDH≤300 U/L group was observed compared with LDH>300 U/L group (D).

Supplementary Table 1 The log-rank analysis of clinical parameters and prognosis

|  | Beta | HR (95% CI) | Z_pval | Logrank_pval | |
| --- | --- | --- | --- | --- | --- |
| CA125 | 1.5797 | 4.85(0.887~26.6) | 0.0685 | 0.0438 |  |
| AST/ALT1.5 | 1.5564 | 4.74(0.849~26.5) | 0.0761 | 0.052 |  |
| AST/ALT2 | 1.3875 | 4(0.792~20.3) | 0.0934 | 0.0705 |  |
| Pathology | -20.731 | 9.92e-10(0~Inf) | 0.999 | 0.0708 |  |
| Symptoms | 20.719 | 9.96e+08(0~Inf) | 0.999 | 0.0753 |  |
| LDH | 1.6925 | 5.43(0.629~46.9) | 0.124 | 0.085 |  |
| Group | 1.1752 | 3.24(0.378~27.8) | 0.284 | 0.257 |  |
| ALT | 0.59239 | 1.81(0.364~8.99) | 0.469 | 0.463 |  |
| AST | 0.59239 | 1.81(0.364~8.99) | 0.469 | 0.463 |  |
| sex | 0.40636 | 1.5(0.174~12.9) | 0.712 | 0.71 |  |
| Age_60 | 0.28258 | 0.754(0.151~3.76) | 0.73 | 0.729 |  |
| AST/ALT1 | 0.27548 | 0.759(0.139~4.15) | 0.751 | 0.75 |  |
| HBV | 0.24976 | 1.28(0.259~6.36) | 0.76 | 0.759 |  |
|  |  |  |  |  |  |
